# Exosomes from hypoxic endothelial cells have increased collagen crosslinking activity through up‐regulation of lysyl oxidase‐like 2

**DOI:** 10.1111/jcmm.12730

**Published:** 2015-11-27

**Authors:** Olivier G. de Jong, Bas W. M. van Balkom, Hendrik Gremmels, Marianne C. Verhaar

**Affiliations:** ^1^ Department of Nephrology and HypertensionUniversity Medical Center UtrechtUtrechtThe Netherlands

**Keywords:** extracellular vesicles, extracellular matrix remodelling, lysyl oxidase

## Abstract

Exosomes are important mediators of intercellular communication. Additionally, they contain a variety of components capable of interacting with the extracellular matrix (ECM), including integrins, matrix metalloproteinases and members of the immunoglobin superfamily. Despite these observations, research on exosome‐ECM interactions is limited. Here, we investigate whether the exosome‐associated lysyl oxidase family member lysyl oxidase‐like 2 (LOXL2) is involved in ECM remodelling. We found that LOXL2 is present on the exterior of endothelial cell (EC)‐derived exosomes, placing it in direct vicinity of the ECM. It is up‐regulated twofold in EC‐derived exosomes cultured under hypoxic conditions. Intact exosomes from hypoxic EC and LOXL2 overexpressing EC show increased activity in a fluorometric lysyl oxidase enzymatic activity assay as well as in a collagen gel contraction assay. Concordantly, knockdown of LOXL2 in exosome‐producing EC in both normal and hypoxic conditions reduces activity of exosomes in both assays. Our findings show for the first time that ECM crosslinking by EC‐derived exosomes is mediated by LOXL2 under the regulation of hypoxia, and implicate a role for exosomes in hypoxia‐regulated focal ECM remodelling, a key process in both fibrosis and wound healing.

## Introduction

Lysyl oxidase‐like 2 (LOXL2) is one of five members of the lysyl oxidase family, consisting of lysyl oxidase (LOX) and lysyl oxidase‐like 1 – 4. Like all LOX family members, LOXL2 facilitates crosslinking of collagens and elastin by catalysing oxidative deamination of lysine residues 1. This process is crucial for giving the extracellular matrix (ECM) its tensile strength and load‐bearing capabilities 2. Although all LOX family members share a high degree of homology, only LOXL2, LOXL3 and LOXL4 possess four scavenger receptor cysteine‐rich domains at their N‐termini 3. The exact function of these domains is yet to be elucidated, but has been suggested to facilitate protein‐protein interactions in the ECM 4. Apart from its extracellular role, intracellular functions of LOXL2 have been reported in epithelial to mesenchymal transition (EMT) and breast cancer metastasis 1, 5.

Lysyl oxidase‐like 2 expression is regulated by hypoxia‐inducible factor 1‐alpha (HIF‐1α), and is up‐regulated in endothelial cells (EC) exposed to low oxygen concentrations 6. Bignon *et al*. showed that LOXL2 produced by EC co‐localizes with, and is required for proper assembly of, collagen IV in the basement membrane 7. Overexpression of LOXL2 in EC resulted in increased migration and tubulogenesis, which was partially inhibited by the specific LOX enzymatic inhibitor β‐aminopropionitrile (BAPN), indicating that the function of EC‐derived LOXL2 at least partially relies on its enzymatic crosslinking activity 7.

In a previous study on EC‐derived exosomes, we observed an approximate twofold increase of LOXL2 abundance when EC were cultured in 2% oxygen 8. Exosomes are extracellular vesicles (EV) that are released by many cell types when the multivesicular body fuses with the plasma membrane and the intraluminal vesicles are released into the extracellular environment 9, 10. Exosomes play a role in intercellular communication through a variety of mechanisms 11, 12. Interestingly, exosomes have also been reported to interact directly with the ECM through integrins and matrix metalloproteinases 13, 14, 15. Given the extracellular function of LOXL2 and the observations regarding interactions of exosomes with the ECM, we hypothesized that exosome‐associated LOXL2 induces ECM crosslinking by deamination of lysine residues of ECM components, under the regulation of hypoxia.

Here, we demonstrate that exosome‐associated LOXL2 is present on the exterior of the vesicles, allowing direct contact and interaction with the ECM. Furthermore, we demonstrate for the first time enzymatic LOX activity of intact exosomes, which is increased upon stimulation of EC with hypoxia. Using a fibroblast collagen gel contraction assay to study crosslinking of collagen I, a similar increase in the activity of EC‐derived exosomes obtained under hypoxic conditions is observed. Lysyl oxidase activity of EC‐derived exosomes was inhibited by lentiviral knockdown of LOXL2, as well as by the LOX enzymatic inhibitor BAPN, and increased by lentiviral overexpression of LOXL2. These data implicate a role for exosomes in hypoxia‐regulated focal ECM remodelling, a key process in both fibrosis and wound healing.

## Materials and methods

### Cell culture

Human microvascular endothelial cells (HMEC‐1) 16, were maintained (up to passage number 28) in MCDB131 medium containing 10% foetal calf serum (FCS), 100 U/ml penicillin and 100 μg/ml streptomycin, 10 mM L‐Glutamine (All from ThermoFisher, Waltham, MA, USA), 50 nm hydrocortisone (Sigma‐Aldrich, St. Louis, MO, USA), and 10 ng/ml rhEGF (Peprotech, Rocky Hill, NJ, USA). Primary adult human dermal fibroblasts (ThermoFisher) were maintained (up to passage 15) in DMEM (ThermoFisher), containing 10% FCS, 100 U/ml penicillin and 100 μg/ml streptomycin. HEK293T cells were cultured in DMEM supplemented with 10% FCS, 100 U/ml penicillin, and 100 μg/ml streptomycin. All cells were cultured at 37°C, 5% CO_2_ and 20% or 2% (where indicated) O_2_. Cells cultured at 2% O_2_ were cultured for 24 hrs in a Invivo2 1000 hypoxic workstation (Baker Ruskinn, Sanford, ME, USA).

### Lentiviral overexpression and knockdown

For lentiviral overexpression the open reading frames encoding for *eGFP* (from pHAGE hSPC‐*eGFP*‐W, 17 or the human LOXL2 (from a pCMV6‐XL5 transient LOXL2 overexpression vector, Origene, Rockville, MD, USA) were cloned into a pHAGE2‐EF1a‐IRES‐PuroR plasmid using the restriction enzymes NotI and BamHI (Promega, Madison, WI, USA) and a Quick Ligation Kit (New England Biolabs, Ipswitch, MA, USA). The LOXL2 open reading frame was fully sequenced to rule out undesirable mutations. Lentiviral knockdown of LOXL2 was achieved using the TRC Human LOXL2 shRNA Gene set (pLKO.1‐PuroR vector containing shRNAs targeting the following sequences: 5′‐ATATTCAGGTTCTCTATCTGG‐3′, 5′‐ATTGTCAAATTTGAACCCAGG‐3′, 5′‐ATGATGTTGTTGGAGTAATCG‐3′, 5′‐ATTCTTCTGGATGTCTCCTTC‐3′) (GE Healthcare, Wauwatosa, WI, USA) (shLOXL2). As a control, pLKO.1‐PuroR without insert was used (shCtrl). For lentiviral production HEK293T cells were transfected with pHAGE2‐EF1a‐IRES‐PuroR, PLP‐VSVG and PSPAX2 plasmids in a 2:1:1 ratio or pLKO.1, pRSV‐REV, pMDL and pVSV‐G in a 3:1:1:1 ratio using 4 μg polyethylenimine per μg of DNA. Culture medium was replaced after 24 hrs, and lentiviral supernatants were harvested after 48 and 72 hrs, cleared from cells by centrifugation, and stored at −80°C until further use. Human microvascular endothelial cells were transfected with lentiviral stocks overnight at 37°C in the presence of 8 μg/ml polybrene (Sigma‐Aldrich). Starting 24 hrs after lentiviral transduction, HMEC‐1 cells were cultured and expanded in the presence of 2.5 μg/ml puromycin for at least 2 weeks.

### Exosome isolation

Exosomes were isolated from conditioned medium by differential ultracentrifugation as described previously 8, 18. Exosome‐free medium (prepared using FCS centrifuged for at least 1 hr at 200,000 × g, followed by 0.2 μm filter‐sterilization) was added to cells grown to ~75% confluence for 24 hrs. During the 24‐hr culturing period cells were exposed to hypoxia (2% O_2_), or ambient oxygen level (20% O_2_). The conditioned medium was centrifuged for 15 min. at 1500 × g to remove cellular debris, after which larger microvesicles were removed by centrifugation for 30 min. at 10,000 × g. Exosomes were isolated by centrifugation for 60 min. at 100,000 × g, and subsequently washed twice by resuspending in PBS and centrifugation for 60 min. at 100,000 × g. Centrifugation was performed using a Beckman LE‐80K centrifuge with SW32‐Ti and SW60‐Ti rotors (Beckman Instruments, Indianapolis, IN, USA).

### Sucrose gradient analysis

Exosomes were resuspended in 250 μl 2.5 M sucrose, 20 mM TRIS HCl pH 7.4 and floated in a SW60 tube for 16 hrs at 190,000 × g using a linear sucrose gradient (2.0–0.25 M sucrose, 20 mM Tris‐HCl, pH 7.4). Gradient fractions (250 μl) were collected from the top and used for subsequent immunoblot analysis.

### Immunoblotting

Exosomes were resuspended in RIPA buffer (Santa Cruz Biotechnology, Dallas, TX, USA), cell lysates were resuspended in 1× Laemmli buffer, and protein concentrations were determined by BCA protein assay (ThermoFisher). Equal protein amounts were subjected to SDS‐PAGE using NuPAGE 4 – 12% Bis‐Tris gradient gels (ThermoFisher), and subsequently transferred to polyvinylidene difluoride (PVDF) membranes (ThermoFisher). Depending on the antibody, PVDF membranes were either blocked in 5% bovine serum albumin (BSA; Roche, Basel, Switzerland) or 5% low‐fat dry milk powder (Campina, Woerden, The Netherlands) in TBS with 0.1% Tween‐20 for 1 hr at RT. polyvinylidene difluoride (PVDF) membranes were incubated with primary antibodies o/n at 4°C, washed in TBS with 0.1% Tween‐20 (TBST), and incubated with horseradish peroxidase (HRP)‐conjugated secondary antibodies in 5% low‐fat dry milk powder in TBST for 1 hr at RT. Membranes were washed in TBS and proteins were visualized using Chemiluminescent Peroxidase Substrate (Sigma‐Aldrich) and imaged on the Molecular Image ChemiDoc XRS system (Bio‐Rad, Hercules, CA, USA). After imaging, PVDF membranes were stripped from their antibodies using ReBlot Plus Mild Antibody Stripping Solution (Millipore, Billerica, MA, USA) for 20 min. at room temperature, blocked and re‐probed for β‐actin as a loading control. The primary antibodies used were β‐actin (cat.no. a5441; Sigma‐Aldrich), Flotillin‐1 (cat.no. sc‐25505; Santa Cruz Biotechnology), CD31 (cat.no. sc‐1505; Santa Cruz Biotechnology), LOXL2 (cat.no. AF2639; R&D Systems, Minneapolis, MN, USA), HIF‐1α (cat.no. NB100‐134; Novus Biologicals, Littleton, CO, USA) and Glyceraldehyde 3‐phosphate dehydrogenase (GAPDH; cat.no. 2118; Cell Signaling Technology, Danvers, MA, USA). Secondary antibodies were HRP‐conjugated swine anti‐rabbit, rabbit anti‐Mouse, and rabbit anti‐goat (All from Dako, Glostrup, Denmark).

### Proteinase K protection assay

Exosomes of approximately 1.5 × 10^7^ cells were isolated by differential ultracentrifugation and resuspended in 20 μl PBS. Samples were incubated in either PBS or 10 μg/ml Proteinase K in PBS, with or without the presence of 1% Triton X‐100, in a final volume of 20 μl per sample for 1 hr at 37°C. The assay was stopped by addition of 20 μl 95°C 2× Laemmli Buffer with 10 mM DTT. After 5 min. incubation at 95°C, samples were used for immunoblot analysis.

### Electron microscopy

Transmission electron microscopy was performed as described 8. After sucrose gradient centrifugation, fractions with a density of 1.10–1.12 g/ml were pooled and centrifuged at 100,000 × g to collect exosomes. The pellet was resuspended in PBS and carbon‐coated Formvar filmed grids were placed on top for 20 min. After three washes with 0.15% glycine in PBS and a 0.1% BSA in PBS wash grids were placed on 5 μl 1% BSA in PBS with 1:100 diluted anti‐LOXL2 antibody for 20 min., washed four times with 0.1% BSA in PBS and incubated on a drop of 1% BSA in PBS containing rabbit anti‐goat polyclonal antibody (Santa Cruz Biotechnology) for 20 min., and were labelled by incubation on PBS containing 1% BSA and 10 nm gold beads coupled to Protein A for 20 min. Vesicles were fixed by incubation in 1% glutaraldehyde in PBS for 5 min. washed twice with PBS and four times with distilled water. Subsequently, grids were placed on ice‐cold 1.8% methylcellulose (25 Ctp)/0.4% Uranyl acetate for 5 min., and vesicles were visualized using a FEI Tecnai 12 (FEI, Hillsboro, OR, USA) transmission electron microscope.

### Fluorescence microscopy

Human microvascular endothelial cells overexpressing *e*GFP were cultured for 48 hrs on Nunc Lab‐Tek II 8‐well chamber slides (ThermoFisher) at standard culturing conditions. Afterwards, cells were fixed for 30 min. at room temperature in 4% PFA‐PBS. Cells were then washed twice with PBS and stained with 1 ug/ml DAPI (ThermoFisher) for 15 min. at room temperature, followed by two more washing steps with PBS. The Lab‐Tek chamber slides were then removed and a cover‐slip was applied using Fluoromount‐G Slide Mounting Medium (ThermoFisher). Fluorescent images were taken using an Olympus BX53 microscope with a DP71 digital camera.

### Fluorometric lysyl oxidase assay

Enzymatic activity of exosomal LOXL2 on 1,5‐diaminopentane (MP Biomedicals, Santa Ana, CA, USA) was determined by measuring horseradish peroxidase type 2 (Sigma‐Aldrich) mediated conversion of Amplex Red (ThermoFisher) to resorufin 19. A substrate mixture [50 mM sodium borate buffer (pH = 8.2), 2.4 M Urea, 20 uM Amplex Red, 20 mM 1,5‐diaminopentane] was mixed with an enzyme mixture [50 mM sodium borate buffer (pH = 8.2) containing 2 U/ml horseradish peroxidase type2] in a 1:1 ratio. Recombinant human LOXL2 (rhLOXL2) (Sino Biological Inc., Beijing, China) was resuspended in the same sodium borate buffer and used for a standard curve. Isolated exosomes were resuspended in PBS, protein concentrations were determined by BCA protein assay, and 5 μg exosomes was analysed per reaction. Assays were performed in a 100 μl volume on a Fluoroskan Ascent FL (ThermoFisher) in kinetics mode with excitation at 544 nm and emission at 570 nm. Measurements were made at a 1 min. interval for 2.5 hrs, at 37°C. The slope of the progress curve was determined over a 30 min. timeframe in the linear region of the curve, and corrected for background activity by deduction of the slope of the same samples analysed in parallel in the presence of 500 μM BAPN (Sigma‐Aldrich).

### Collagen gel contraction assay

Adult human dermal fibroblasts cells were cultured for 48 hrs in serum‐free medium (DMEM containing 100 U/ml penicillin and 100 μg/ml streptomycin), and collected by incubation with TrypLE Express (ThermoFisher) for 5 min. at 37°C. Cells were subsequently pooled using serum‐free medium and pelleted by centrifugation for 8 min. at 400 × g. After resuspension in serum‐free medium, cells were added to a mixture of Rat Tail Collagen I (ThermoFisher), 2× modified eagle medium (MEM) (Life Technologies, Carlsbad, CA, USA), sterilized Milli‐Q water and 1N NaOH, with a final concentration of 1 mg/ml Collagen I, 1× MEM, 0.2 million cells/ml, and physiological pH. After addition of 20 μg/ml exosomes in PBS, 2 μg/ml rhLOXL2, 500 μM BAPN, or a similar volume of PBS for control conditions, 0.5 ml gel matrices were made in 24‐well plate wells. Gels were allowed to set for 30 min. at 37°C, and were then transferred to 12‐well plate wells containing 1 ml 1× MEM. Collagen gels were incubated for 48 hrs at 37°C, 5% CO_2_, and pictures were taken every 12 hrs using the Molecular Image ChemiDoc XRS system (Bio‐Rad). Gel surfaces were determined with ImageJ software using the polygon selection tool, followed by an area measurement. Collagen contraction was calculated using the following equation:

$$\text{Collagen contraction}=1-\left(\frac{\text{Collagen gel size at}\;t=36\;\text{hrs}}{\text{Collagen gel size at}\;t=0\;\text{hrs}}\right)$$

### Statistical analyses

All data are expressed as mean ± SD. Student's *t*‐test or one‐way anova with Sidak's multiple comparisons post‐hoc analysis was used to compare groups, *P* < 0.05 was considered significant.

## Results

### Hypoxia results in increased exosomal LOXL2 abundance

**Figure 1:**
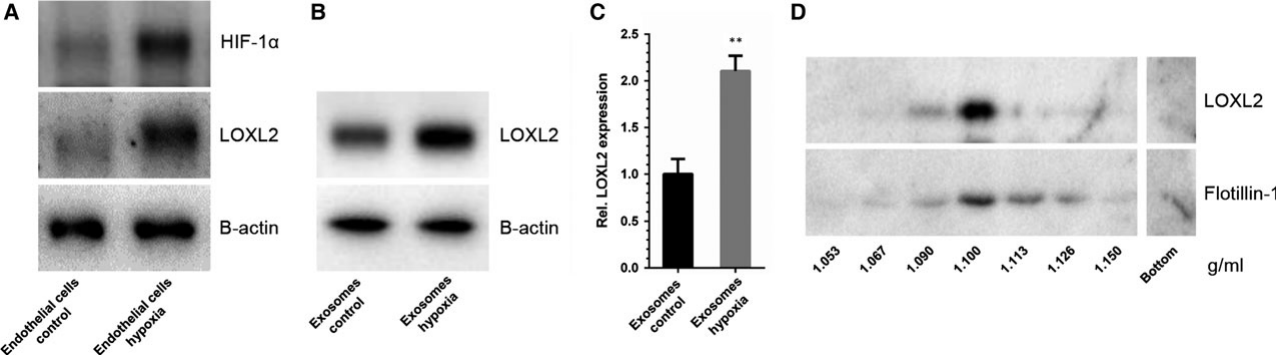
LOXL2 abundance in EC‐derived exosomes is increased in hypoxia. (**A**) Immunoblots of endothelial cells for HIF‐1α (control for hypoxia, top panel), LOXL2 (middle panel) and β‐actin (loading control, lower panel). (**B**) Immunoblots of EC‐derived exosomes for LOXL2 (upper panel), and β‐actin (loading control, lower panel). (**C**) Densitometric quantification of relative LOXL2 protein abundance in control and hypoxic EC‐derived exosomes (*n* = 4 ± SD, Student's *t*‐test; **P < 0.01). (**D**) Immunoblots of sucrose density gradient samples of EC‐derived exosomes for LOXL2 (upper panel) and exosome‐marker Flotillin‐1 (lower panel).

We have previously shown that cellular stress is represented in EC‐derived exosomes 8. One of the exosomal proteins that was increased upon cellular hypoxic stress was the LOX family member LOXL2. To quantify the observed increase in exosomal LOXL2 abundance, HMEC‐1 were cultured in control‐ and hypoxic conditions, as was confirmed by immunoblot analysis for HIF‐1α and LOXL2 (Fig. 1A), after which exosomes from these cells were isolated and immunoblotted for LOXL2, using β‐actin, which we previously showed to remain unchanged upon hypoxic stimulation in EC‐derived exosomes 8, as a loading control (Fig. 1B). Densitometric analysis showed a twofold increase of LOXL2 (Fig. 1C), in line with the previously performed quantitative proteomics analysis 8. To further illustrate that LOXL2 is indeed associated with exosomes, sucrose density gradient and subsequent immunoblot analysis were performed, showing that LOXL2 and the exosome marker Flotillin‐1 are both detected at a density around 1.10 g/ml (Fig. 1D).

### Exosomal LOXL2 is located on the exterior of exosomes

**Figure 2:**
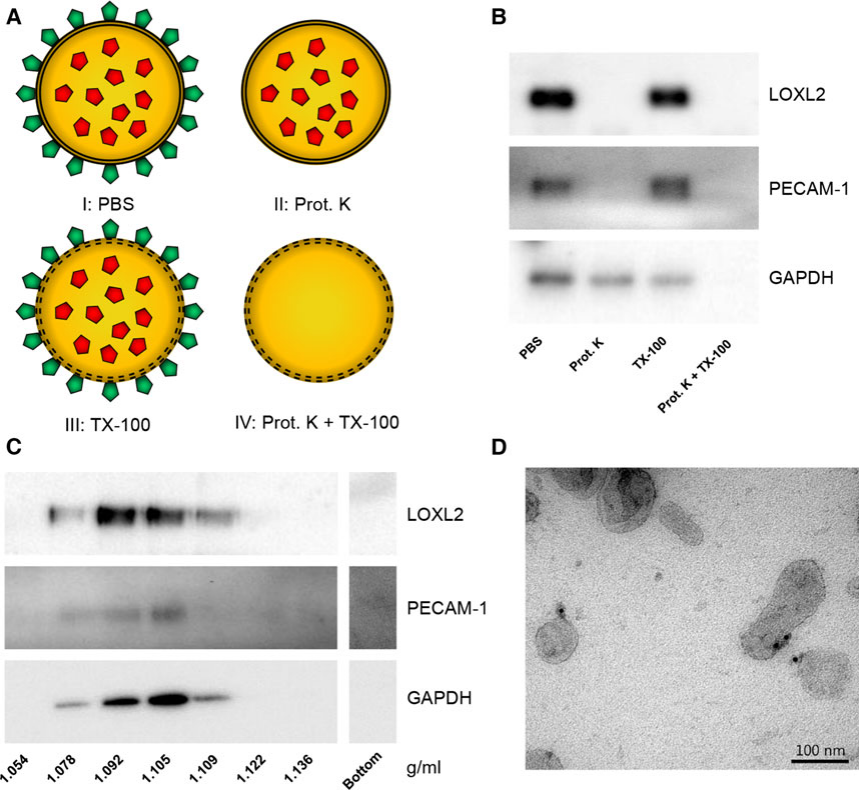
Exosome‐associated LOXL2 is present on the exterior of exosomes. (**A**) A schematic representation of the proteinase K protection assay. External proteins are depicted in green, and internal proteins in red. The lipid bi‐layer is represented by a double black line. (I) All exosomal proteins remain present in PBS; (II) Proteins exposes at the exosome surface are removed by proteinase K digestion; (III) All exosomal proteins remain present in TX‐100‐treated exosomes; (IV) Proteinase K treatment of TX‐100 permeabilized exosomes digests exterior and interior proteins. (**B**) Intact EC‐derived exosomes were incubated in PBS for 1 hr at 37°C in the presence of 10 μg/ml proteinase K, 0.1% Triton X‐100, or both (indicated). (**C**) Immunoblots showing that all proteins tested in the proteinase K protection assay are associated with exosomes. (**D**) Electron micrograph confirming of the exterior localization of LOXL2 on EC‐derived exosomes (10 nm gold: LOXL2).

To determine the location of LOXL2, intact EC‐derived exosomes were treated with proteinase K, with or without TX‐100 to permeabilize exosome membranes (Fig. 2A), using exosomal platelet endothelial cell adhesion molecule (PECAM‐1) and GAPDH as controls for membrane and cytosolic proteins, respectively (Fig. 2B). If exosomes were left untreated, immunoblotting analysis showed presence of LOXL2, PECAM‐1 (membrane protein control) and GAPDH (cytoplasmic protein control), all associated with exosomes as shown by sucrose density gradient analysis (Fig. 2C). Proteinase K treatment digested both LOXL2 and PECAM‐1, whereas GAPDH, which is present in the interior of exosomes and thus protected by the lipid bi‐layer, was not digested. Permeabilization of the exosome membrane by TX‐100 makes GAPDH accessible and thus susceptible to proteinase K, resulting in its degradation. This demonstrates that exosomal LOXL2 is present on the exterior of the vesicle membrane, placing it in the same compartment as the ECM. These findings were confirmed by electron microscopy (Fig. 2D).

### Hypoxia results in increased lysyl oxidase and collagen crosslinking activity of EC‐derived exosomes

**Figure 3:**
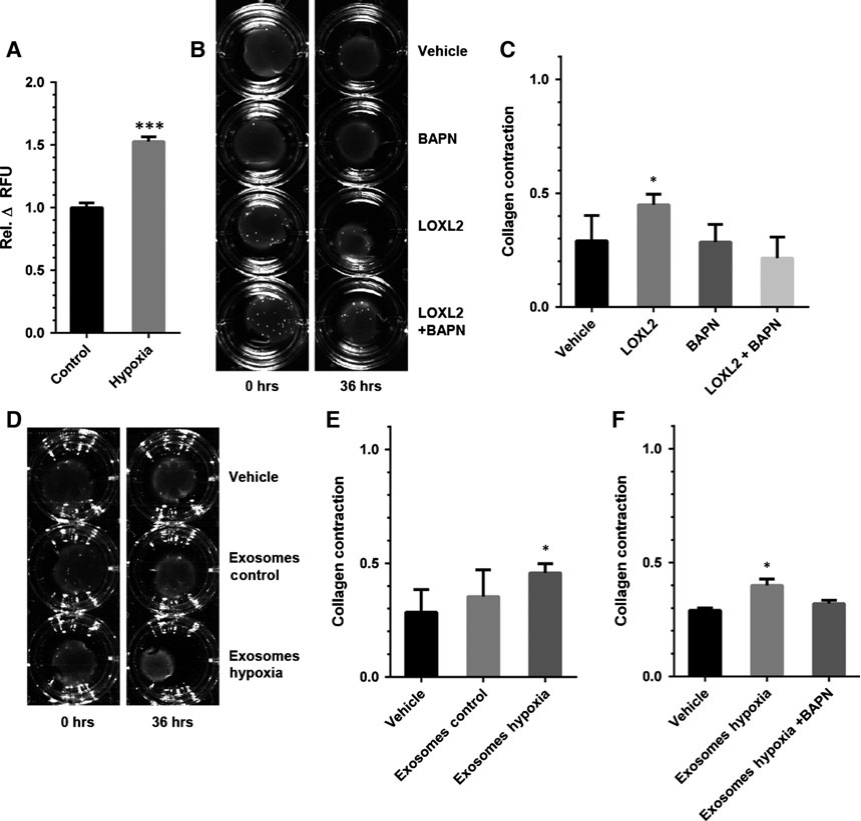
Lysyl oxidase activity is increased in exosomes from hypoxic EC. (**A**) Lysyl oxidase activity analysis of intact exosomes from EC cultured at 20% (Control) and 2% O_2_ (Hypoxia) (*n* = 4 ± SD, Student's *t*‐test). (**B**) Representative images of collagen gel contraction assays using buffer (Vehicle), rhLOXL2 (LOXL2), the enzymatic inhibitor β‐aminopropionitrile (BAPN) and the combination of both (LOXL2 + BAPN) at *t* = 0 hr and *t* = 36 hrs, and (**C**) quantification of gel contraction after 36 hrs for these conditions (*n* = 3 ± SD, anova). (**D**) Representative images of collagen gel contraction assays using control buffer (Vehicle), exosomes from EC cultured under control (Exosomes Control) and hypoxic (Exosomes Hypoxia) conditions, and (**E**) quantification of gel contraction after 36 hrs for these conditions (*n* = 3 ± SD, anova). (**F**) Quantification of collagen gel contraction assays using control buffer (Vehicle), exosomes from EC cultured under hypoxic conditions (Exosomes Hypoxia) and exosomes from EC cultured under hypoxic conditions with BAPN (Exosomes Hypoxia +BAPN) after 36 hrs. *P < 0.05; ***P < 0.001.

Using an *in vitro* fluorometric LOX activity assay with 1,5‐pentanediamine as a substrate 19, the rate of LOXL2‐catalysed oxidative deamination was measured. Exosomes from EC cultured in hypoxic conditions showed a 1.5‐fold increase of LOX activity as compared to exosomes derived from EC cultured in control conditions (Fig. 3A).

To investigate exosomal LOXL2‐mediated crosslinking of collagen, we used a collagen gel contraction assay in which increased ECM stiffness as a result of collagen crosslinking by LOXL2 results in fibroblast activation and subsequent gel contraction (Fig. 3B) 20. In the presence of rhLOXL2, collagen gel contraction was increased, and this effect was fully inhibited by the LOX crosslinking inhibitor BAPN fumarate (Fig. 3C). We then analysed the effect of EC‐derived exosomes in the collagen gel contraction assay (Fig. 3D). Exosomes derived from EC cultured in hypoxic conditions induced an increase in collagen contraction after 36 hrs (Fig. 3E), which was abolished by addition of BAPN (Fig. 3F).

### LOXL2 overexpression results in increased exosomal lysyl oxidase activity

**Figure 4:**
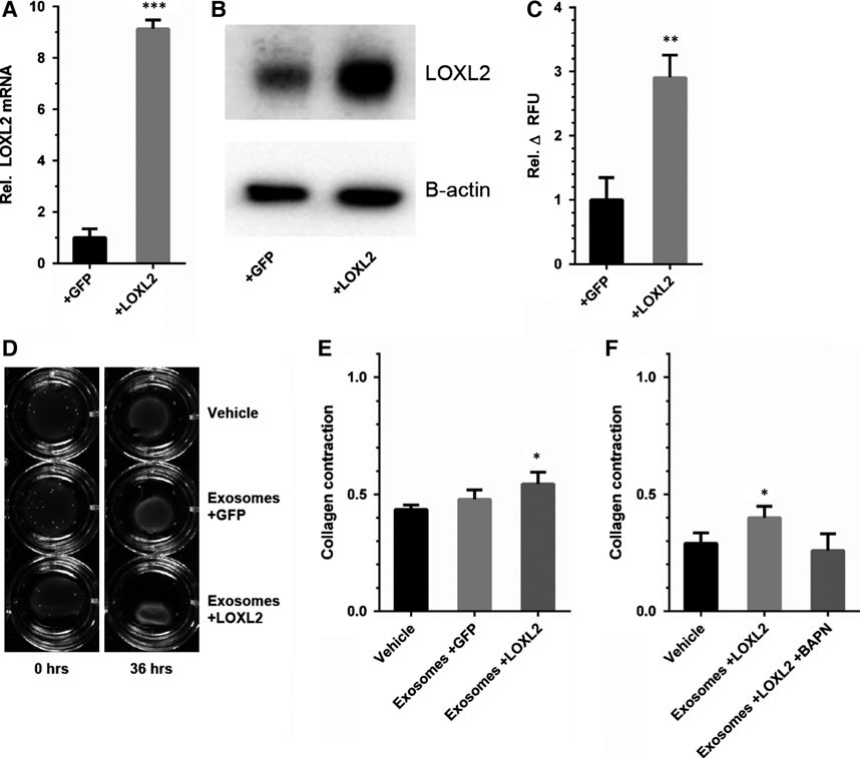
LOXL2 overexpressing EC produce LOXL2‐rich exosomes that potently crosslink collagen. (**A**) LOXL2 mRNA expression is increased in LOXL2‐overexpressing EC (+LOXL2) compared to control‐infected EC (+GFP) (*n* = 3 ± SD, Student's *t*‐test). (**B**) LOXL2 protein expression is increased in LOXL2 overexpressing EC‐derived exosomes (**C**) *In vitro* lysyl oxidase activity analysis of exosomes from control‐infected (+GFP) and LOXL2 overexpressing (+LOXL2) EC (*n* = 3 ± SD, Student's *t*‐test). (**D**) Collagen gels containing control buffer (Vehicle) and exosomes from control‐infected (Exosomes+GFP) and LOXL2 overexpressing (Exosomes+LOXL2) EC imaged at different time points and (**E**) quantification of gel contraction after 36 hrs. (**F**) Quantification of collagen gel contraction assays using control buffer (Vehicle), exosomes from EC overexpressing LOXL2 (Exosomes +LOXL2) and exosomes from EC overexpressing LOXL2 with BAPN (Exosomes + LOXL2 + BAPN) after 36 hrs. (*n* = 3 ± SD, anova). *P < 0.05; **P < 0.01; ***P < 0.001.

Human microvascular endothelial cells were transfected by with a pHAGE2 lentiviral vector containing a LOXL2 open reading frame (+LOXL2), or containing *eGFP* (+GFP) as a control. LOXL2 overexpression was confirmed by qPCR, showing an eightfold increase of LOXL2 mRNA (Fig. 4A), and immunoblotting confirmed 2.7‐fold increase of LOXL2 protein abundance in exosomes (Fig. 4B), which corresponded well with the approximately threefold higher *in vitro* LOX activity compared to control (+GFP)‐EC derived exosomes (Fig. 4C). In control (+GFP) cells *e*GFP expression was confirmed by fluorescence microscopy (Fig. S1). LOXL2‐high exosomes were also used for a collagen gel contraction assay (Fig. 4D). Similar to exosomes derived from EC cultured in hypoxic conditions, LOXL2‐high exosomes increased collagen gel contraction, whereas GFP‐high exosomes did not (Fig. 4E), and this effect was abolished by addition of BAPN (Fig. 4F).

### EC‐exosome‐stimulated collagen crosslinking activity is abolished by LOXL2 knockdown

**Figure 5:**
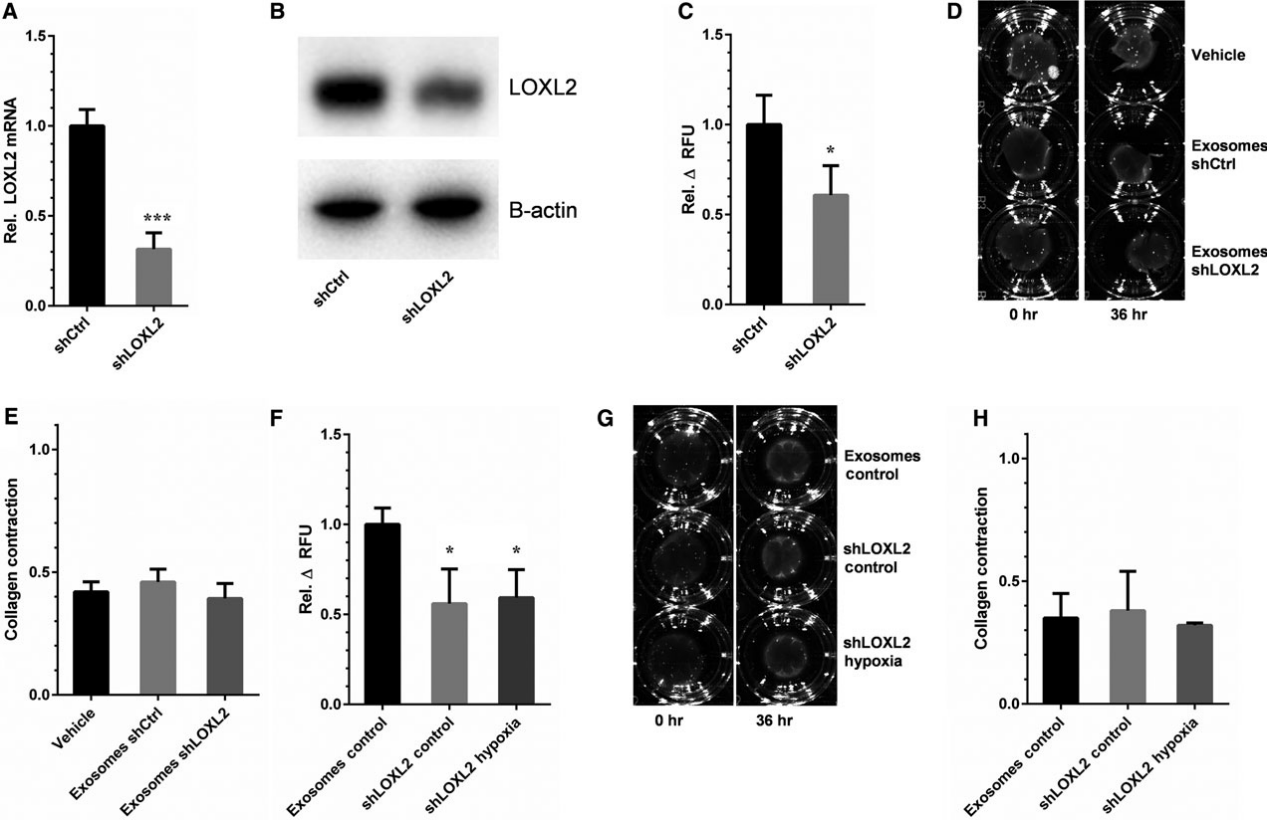
LOXL2 is responsible for increased collagen crosslinking activity in hypoxic EC‐derived exosomes. (**A**) LOXL2 mRNA expression is decreased in shLOXL2‐transfected EC (shLOXL2) compared to control‐infected EC (shCtrl) (*n* = 7 ± SD, Student's *t*‐test). (**B**) LOXL2 protein expression is decreased in shLOXL2‐transfected EC‐derived exosomes. (**C**) Analysis of lysyl oxidase activity in exosomes from control (shCtrl) and LOXL2 knock‐down (shLOXL2) (*n* = 3 ± SD, Student's *t*‐test). (**D**) Collagen gels comparing control buffer (Vehicle), and exosomes from control (Exosomes shCtrl) and LOXL2 knock‐down (Exosomes shLOXL2) EC, and (**E**) quantification of gel contraction after 36 hrs for these conditions (*n* = 3 ± SD, anova). Collagen crosslinking activity of exosomes from control and LOXL2 knock‐down EC at 20% (Exosomes Control and shLOXL2 Control) and 2% O_2_ (shLOXL2 Hypoxia) was assessed using (**F**) the *in vitro* lysyl oxidase assay and (**G** and **H**) the collagen gel contraction assay (*n* = 3 ± SD, anova). *P < 0.05; ***P < 0.001.

To confirm whether LOXL2 is responsible for the activity of EC‐derived exosomes observed in LOX and collagen gel contraction assays, HMEC‐1 cells were transfected by lentivirus with a pLKO.1 vector containing a mixture of four short hairpin RNAs (shRNAs) against LOXL2 (shLOXL2), or with a pLKO.1 vector without any shRNA inserted (shCtrl). A 70% LOXL2 knockdown in EC was verified by both qPCR (Fig. 5A), and immunoblot analysis (Fig. 5B). Additionally, LOXL2‐low exosomes showed a 40% decreased LOX activity in a fluorometric assay as compared to shCtrl‐derived exosomes (Fig. 5C). In a collagen gel contraction assay (Fig. 5D), exosomes from either shCtrl‐ or shLOXL2‐EC did not increase contraction (Fig. 5E).

Finally, to determine whether the increased activity observed in hypoxic exosomes was LOXL2‐mediated, LOX activity of LOXL2‐low exosomes isolated in normoxic (control) and hypoxic conditions were analysed (Fig. 5F). LOXL2 knockdown resulted in a 40% reduced LOX activity in EC‐derived exosomes, which did not increase upon hypoxic stimulation of their producing cells. In a collagen gel contraction assay LOXL2 knockdown exosomes isolated in control and hypoxic conditions (Fig. 5G) did not significantly affect gel contraction (Fig. 5H). Altogether, these findings show that ECM crosslinking by EC‐derived exosomes is mediated by LOXL2, under the regulation of hypoxia.

## Discussion

Lysyl oxidase‐like 2 was first described in 1998 21, and has been linked to ECM remodelling, angiogenesis, cell proliferation, migration, transcription regulation, fibroblast activation, EMT and metastatic niche formation through a variety of pathways 1, 5, 7, 20, 22, 23, 24, 25. LOXL2 also regulates a number of processes through intracellular signalling activity. For instance, Peinado *et al*. postulated that LOXL2 regulates EMT by interacting with the EMT‐regulator Snail, inhibiting glycogen synthase kinase 3β‐mediated phosphorylation, and subsequent degradation, thereby inducing EMT 26. Additionally, LOXL2 has also been shown to suppress E‐cadherin in epithelial cells by deamination of a specific lysine residue in histone H3 22.

Effects of LOXL2 on processes like angiogenesis, fibroblast activation and pre‐metastatic niche formation on the other hand mainly depend on its extracellular role in ECM crosslinking 7, 20, 25. Regulatory roles for exosomes have been described in all these processes. Endothelial cell‐derived exosomes increase angiogenesis by both regulating Notch‐dependent tip cell formation and repression of senescence of ECs 18, 27. Upon hypoxic stimulation, epithelial cell‐derived exosomes are capable of activating fibroblasts by transformning growth factor‐β signalling 28, and tumour exosomes affect the pre‐metastatic niche by transfer of miRNAs 29. Interestingly, LOXL2 has also been found in proteomic analysis of extracellular vesicles of squamous carcinoma cells 30 and human umbilical vein endothelial cell (HUVEC)‐derived exosomes 31, and both LOXL2 and LOX were found in exosomes isolated from glioblastoma cells 32, 33. However, a potential role of exosomal LOXL2 in the aforementioned processes has thus far remained uninvestigated.

Here, we describe for the first time that exosomal LOXL2 is enzymatically active and can catalyse the crosslinking of collagen in the ECM. Although our assays measure total activity of all LOX family members the HMEC‐1 derived exosomes make an excellent model system to investigate the role of exosomal LOXL2 as our previous proteomics analysis revealed that LOXL2 is the only member of the LOX family that can be detected in HMEC‐1 derived exosomes 8. Using this model system, we show a twofold increase of LOXL2 abundance in EC‐derived exosomes. Since LOXL2 expression is at least in part regulated by HIF‐1α 34, increased expression of LOXL2 as a response to hypoxia is not unexpected. The presence and upregulation of LOXL2 on the surface of EC‐derived exosomes was particularly interesting, since LOXL2 is generally known as a soluble secreted enzyme 35. A yeast‐two hybrid screen performed by Hollosi *et al*. revealed that several ECM proteins may interact with LOXL2, including fibronectin 1. Proteomic analysis of HMEC‐1 derived exosomes revealed presence of a large number of ECM proteins, including fibronectin, which was also up‐regulated twofold in exosomes in hypoxic conditions 8. Additionally, it has been shown that proteolytic activation of LOX is regulated through interaction with fibronectin 36. These findings suggest that exosomal LOXL2 is present on the exterior of exosomes as a result of interactions with exosome‐associated ECM components.

Despite a twofold increase of LOXL2 abundance in hypoxic conditions, we observed an only approximately 1.5‐fold increase of LOX activity. Given the complex nature of exosomes, it is possible that they contain several components that might interfere with the *in vitro* assays used to measure LOX activity. For example, EC‐derived exosomes also contain Semenogelin, a reactive oxygen species production inhibitor 37 that is also up‐regulated in HMEC‐1 derived exosomes in hypoxic conditions 8. Since the Amplex Red based fluorometric assay used is based on reactive oxygen species production as a result of deamination of the substrate 19, it is possible that presence of such exosomal proteins results in an underrepresentation of enzymatic activity. However, in both assays, the observed increase in LOX activity under hypoxic conditions was reduced by LOXL2 knockdown in the exosome‐producing cells, confirming that LOXL2 was indeed responsible for the observed LOX activity.

Altogether, these data indicate that crosslinking components of the ECM can be added to the functional repertoire of exosomes. Given the known role of LOXs in processes like angiogenesis, fibrosis and pre‐metastatic niche formation these findings merit additional studies on exosomal LOX activity, as players in healing responses and in aberrant ECM remodelling in pathologies, as well as therapeutic applications and targets.

## Conflicts of interest

The authors confirm that there are no conflicts of interest.

## Author contributions

O.G.d.J. and B.W.M.v.B. designed the study and wrote the paper. H.G. performed technical assistance and contributed to the preparation of the manuscript. M.C.V. supervised the project and contributed to the preparation of the manuscript. All authors reviewed and approved the final version of the manuscript.

## Supporting information


**Figure S1** Expression of *e*GFP in control +GFP endothelial cells is confirmed by fluorescence microscopy.
