# Supplementary figures and images for: Exosomes from hypoxic endothelial cells have increased collagen crosslinking activity through up‐regulation of lysyl oxidase‐like 2
Source: J Cell Mol Med. 2015 Nov 27;20(2):342–50. doi: 10.1111/jcmm.12730 (PMC4727569; doi:10.1111/jcmm.12730)

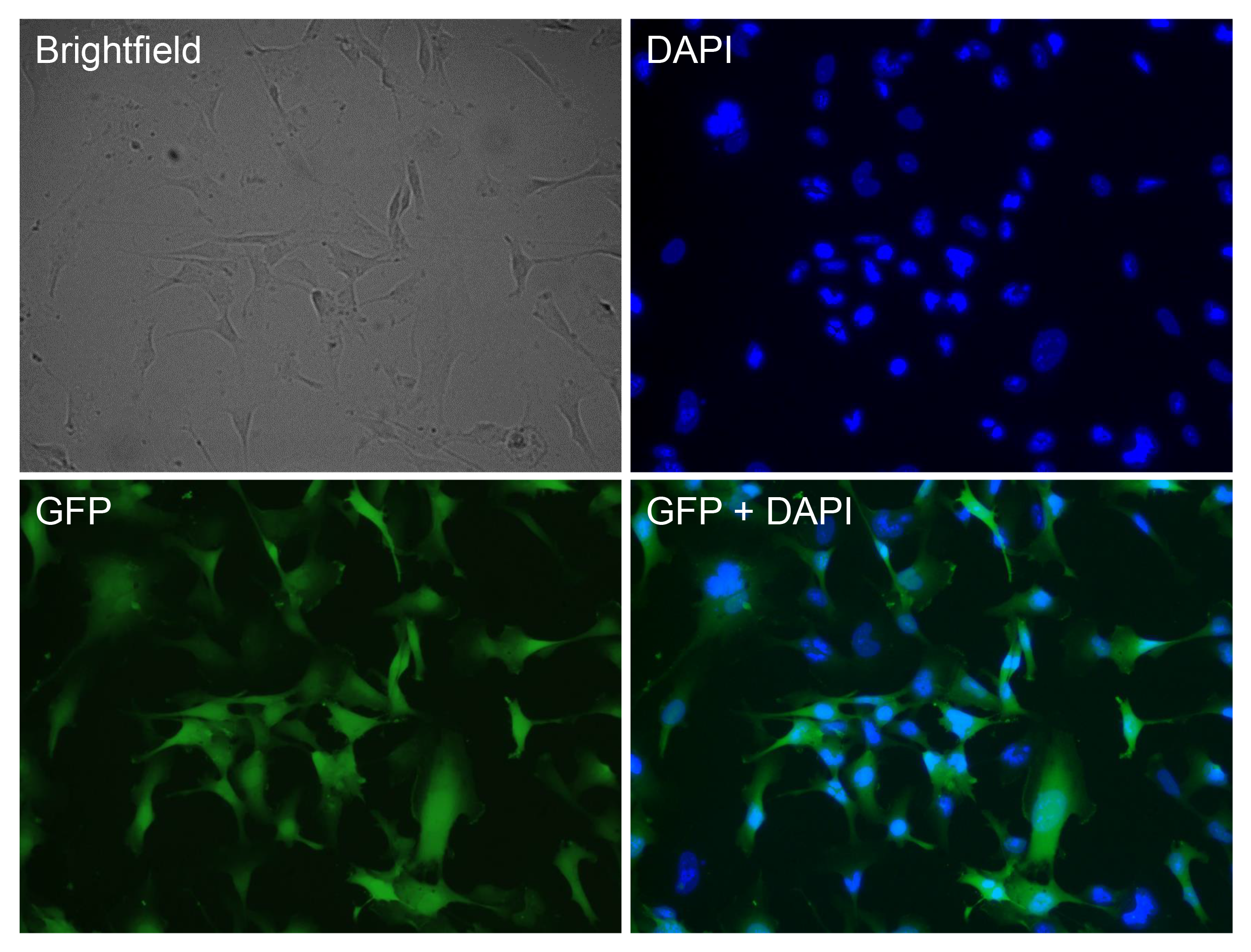

Supplement: Supplementary file 1 — Figure S1 Expression of eGFP in control +GFP endothelial cells is confirmed by fluorescence microscopy. [file JCMM-20-342-s001.tif]
